# Small Leucine-Rich Proteoglycans in Skin Wound Healing

**DOI:** 10.3389/fphar.2019.01649

**Published:** 2020-01-28

**Authors:** Xiaoxiao Pang, Nuo Dong, Zhong Zheng

**Affiliations:** ^1^ Chongqing Key Laboratory of Oral Diseases and Biomedical Sciences, Stomatological Hospital of Chongqing Medical University, Chongqing, China; ^2^ Division of Growth and Development, School of Dentistry, University of California, Los Angeles, Los Angeles, CA, United States

**Keywords:** skin, skin wound healing, small leucine rich proteoglycans, extracelluar matrix, fibromodulin, decorin, biglycan, lumican

## Abstract

Healing of cutaneous wounds is a complex and well-coordinated process requiring cooperation among multiple cells from different lineages and delicately orchestrated signaling transduction of a diversity of growth factors, cytokines, and extracellular matrix (ECM) at the wound site. Most skin wound healing in adults is imperfect, characterized by scar formation which results in significant functional and psychological sequelae. Thus, the reconstruction of the damaged skin to its original state is of concern to doctors and scientists. Beyond the traditional treatments such as corticosteroid injection and radiation therapy, several growth factors or cytokines-based anti-scarring products are being or have been tested in clinical trials to optimize skin wound healing. Unfortunately, all have been unsatisfactory to date. Currently, accumulating evidence suggests that the ECM not only functions as the structural component of the tissue but also actively modulates signal transduction and regulates cellular behaviors, and thus, ECM should be considered as an alternative target for wound management pharmacotherapy. Of particular interest are small leucine-rich proteoglycans (SLRPs), a group of the ECM, which exist in a wide range of connecting tissues, including the skin. This manuscript summarizes the most current knowledge of SLRPs regarding their spatial-temporal expression in the skin, as well as lessons learned from the genetically modified animal models simulating human skin pathologies. In this review, particular focus is given on the diverse roles of SLRP in skin wound healing, such as anti-inflammation, pro-angiogenesis, pro-migration, pro-contraction, and orchestrate transforming growth factor (TGF)β signal transduction, since cumulative investigations have indicated their therapeutic potential on reducing scar formation in cutaneous wounds. By conducting this review, we intend to gain insight into the potential application of SLRPs in cutaneous wound healing management which may pave the way for the development of a new generation of pharmaceuticals to benefit the patients suffering from skin wounds and their sequelae.

## Introduction

**Figure 1:**
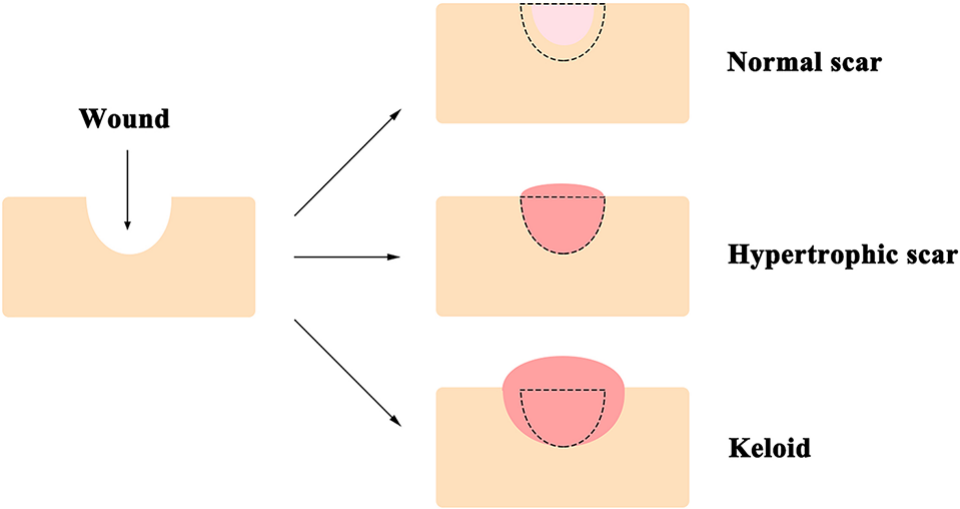
A diagram of typical appearances of normal scar, hypertrophic scar, and keloid. Unlike that the normal scar is often smaller than the original wound, keloids extend beyond the edge of the original wound, while hypertrophic scars are restricted to the wound borders. The black dotted line demarcates the area of the original wound.

The skin, comprised of the epidermis, dermis, and deeper subcutaneous tissue, is the largest organ of the body, and functions as the first line of defense from external assaults (Proksch et al., 2008). Surgery and trauma in adults often result in wounds, which can cause the formation of refractory scars [i.e., hypertrophic scars and keloids, which are specific to humans (Baker et al., 2009)] with significant functional and psychological consequences (Bayat et al., 2003) that reduce the quality of life of individuals (Brown et al., 2008). Compared to the normal scars that can be much smaller than the original wound, keloids are defined as pathologic scars that extend beyond the area of the original wound, while hypertrophic scars are restricted to the wound borders (**Figure 1**) (Atiyeh et al., 2005; Baker et al., 2009; Naylor and Brissett, 2012). Consequently, annual spending on managing unwanted scarring exceeds $20 billion in the United States (Block et al., 2015). Local corticosteroid injection and radiation therapy are the current standards of care for patients who suffer from scar formation (Tziotzios et al., 2012); however, neither method shows consistent efficacy and often results in undesirable, sometimes severe, side effects (Gauglitz et al., 2011). For instance, local corticosteroids injection is known to cause reduced wound strength with increased risks of dehiscence, pigmentation changes, granulomas, and skin atrophy (Chang and Ries, 2001; Bayat et al., 2003); while radiation therapy is associated with growth inhibition, decreased wound strength, and increased long-term cancer risks (Haubner et al., 2012).

To date, several anti-scarring products targeting growth factors involved in the cutaneous wound healing process are being, or have been, tested in clinical trials for wound healing management. These include Juvista™ [recombinant transforming growth factor (TGF)β3, traditionally considered as an “antifibrotic TGFβ isoform”; clinicaltrials.gov: NCT00742443], interleukin (IL)10 (clinicaltrials.gov: NCT00984646) (Kieran et al., 2013; Kieran et al., 2014), DSC127 (NorLeu3-angiotensin; clinicaltrials.gov: NCT01830348), siRNA (RXI-109; clinicaltrials.gov: NCT02030275) and antisense oligonucleotides (EXC 001; clinicaltrials.gov: NCT01038297) that downregulate the expression of connective tissue growth factor (CTGF). However, most of these products failed to demonstrate efficacy in human trials. For instance, Juvista™ failed in phase III clinical trial in 2011 (McKee, 2011); Derma Sciences reported to stop all development work with DSC127 in scar reduction in 2015 (Levin, 2015); IL10 showed no efficacy of scar reduction in humans of African continental ancestral origin (Kieran et al., 2014); and clinical trials appear to have been halted for EXC001 (Pfizer, 2011) as there have been no public updates since 2012; Relatively, RXI-109 seems to have some benefits on the visual appearance of scar tissue in phase II clinical trials, but it requires multiple post-surgery injections, which bring higher therapeutic costs and increase the patient’s suffering (Galiano, 2015). As a consequence, no drugs have been officially approved for the prevention and reduction of cutaneous scarring.

The extracellular matrix (ECM), composed of numerous macromolecules, not only functions as the critical structural components but also plays essential roles in modulating vital cellular processes, such as adhesion (Jian et al., 2013; Desseaux and Klok, 2015; Shih et al., 2016), migration (Estrach et al., 2011; Daley and Yamada, 2013; Jian et al., 2013; Scarpa and Mayor, 2016; Zheng et al., 2017), proliferation (Wight et al., 1992; Leiton et al., 2015; Cheng et al., 2016), differentiation (Jian et al., 2013; Hoshiba et al., 2016; Zheng et al., 2017), apoptosis (Ii et al., 2006; Oskarsson et al., 2015; Zhang et al., 2015), and cell fate determination (Bi et al., 2007; Zheng et al., 2012; Li et al., 2016; Zheng et al., 2019). Consequently, the ECM-based pharmacotherapeutics have been considered for treating fibrotic diseases (Ye et al., 2007), osteoarthritis (Clegg et al., 2006), osteoporosis (Stoch and Wagner, 2008), and malignancies (McKenzie, 2007). The most abundant ECM protein in connective tissues, collagen, forms the highly organized, three-dimensional macrostructure of the healthy skin (Ruszczak, 2003; Davison-Kotler et al., 2019). The initial formation and maintenance of normal, healthy collagenous matrix alignment require proteoglycans (PGs) (Chen and Birk, 2013), which are another broadly distributed component of the ECM in connective tissues to provide resilience, viscoelasticity, and a suitable environment for cellular function and development (Iozzo and Schaefer, 2015). Consisting of a core protein covalently attached with one or more glycosaminoglycan (GAG) chains, PGs play a pivotal role in the proper alignment of fibrous and elastic components in the skin and control the bioavailability of several growth factors in the ECM surrounding cells to stimulate the skin turnover and repair (Mary and James, 2015). Based on their structure, location, and properties, PGs can be divided into 4 classes: intracellular PGs, basement membrane PGs, cell-surface PGs, and extracellular PGs (Vynios, 2014; Iozzo and Schaefer, 2015). In this review we are primarily concerned with the extracellular PGs, which are known to play a role in skin wound healing. For instance, a large, aggregating and water-retaining extracellular PG, versican, is widely detected in the skin (Carrino et al., 2011). Versican accumulation in the pericellular matrix leads to the fibroblast-myofibroblast transition in the dermis by knocking out a versican-degrading protease [ADAM metallopeptidase with thrombospondin type 1 motif (ADAMTS)5] (Hattori et al., 2011). This indicates that versican accumulation may be beneficial for skin wound healing since the fibroblast-myofibroblast transition is pivotal for wound contraction. Aggrecan is another extracellular PG that was initially found in the cartilage and is absent in normal skin but accumulates in scar tissue (Velasco et al., 2011; Vynios, 2014; Mary and James, 2015). Aggrecan accumulation may hinder cell migration to the wound and prevent the transition of fibroblast progenitor cells to mature fibroblasts (Velasco et al., 2011). These studies suggest that aggrecan may be a potential target for reducing scar formation.

**Figure 2:**
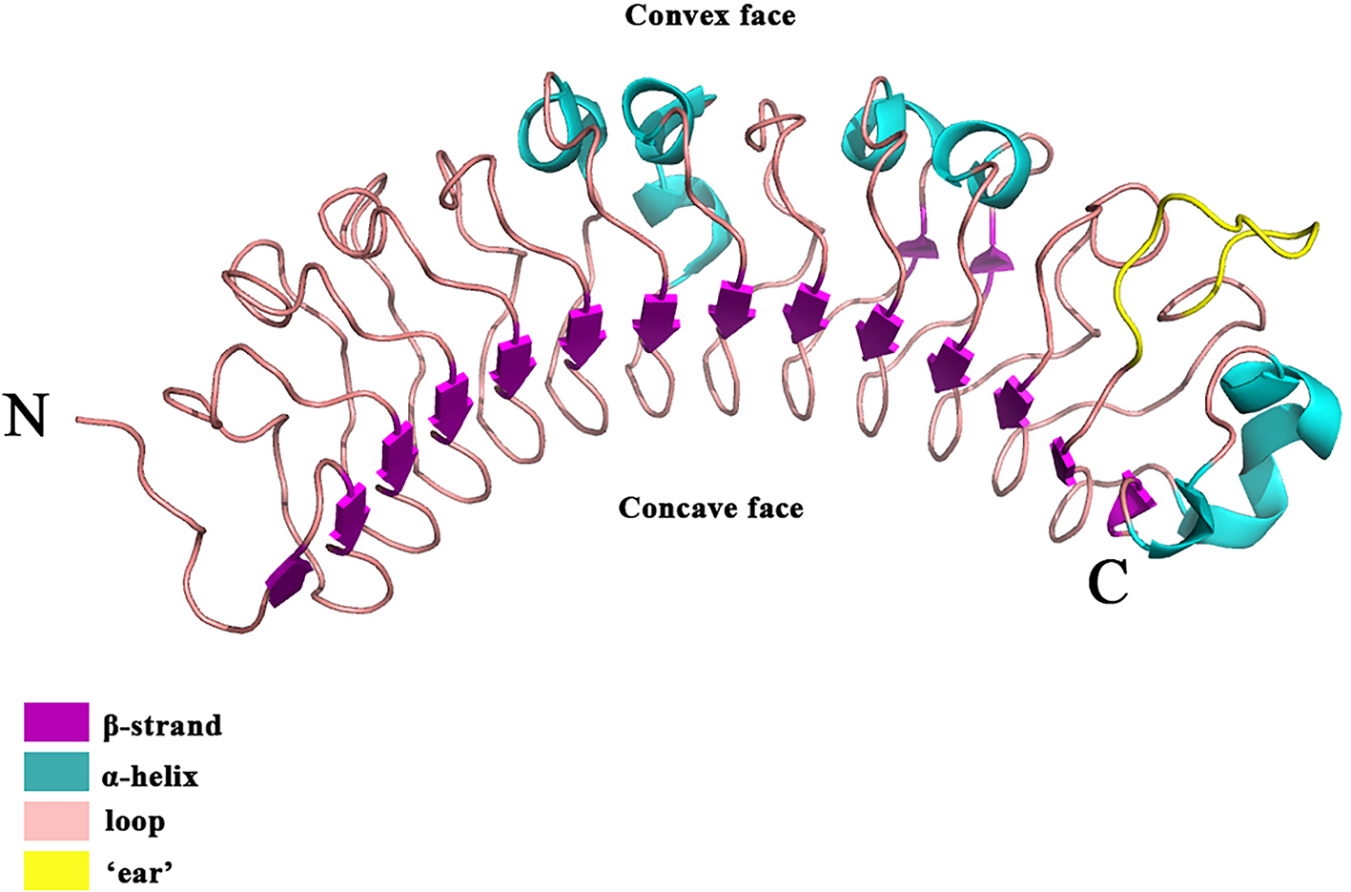
The crystal structure of the DCN. DCN is the archetypal SLRP [the structure was retrieved from Protein Data Bank (PDB), ID: 1XKU] (Scott et al., 2004). DCN is a single-domain structure with a righthanded, curved solenoid fold characteristic of LRR proteins. The long β-sheet that forms the inner, concave face is comprised of 14 β-strands. The penultimate LRR that extends laterally from the main body of the molecule is referred to as the ‘ear’ (yellow) repeat, which is thought to be a distinctive feature of the SLRP family.

Besides versican and aggrecan, small leucine-rich proteoglycans (SLRPs), constitute another large family of extracellular PGs (Pietraszek-Gremplewicz et al., 2019) that play a pivotal role in collagen fibril growth, fibril organization, and ECM assembly in healthy skin (Merline et al., 2009; Chen and Birk, 2013). A typical SLRP has a core protein of 40–60 kDa with 10–12 leucine-rich repeat (LRR) motifs (Iozzo, 1999). Each LRR motif contains 20–29 amino acids, in which an 11-amino acid hallmark, LXXLXLXXNXL (X being any amino acid) can be identified (Iozzo, 1998; McEwan et al., 2006; Bella et al., 2008). Each LRR motif generally forms a curved conchoid structure in which LXXLXLXXNXL builds a β-strand, while β-strands from the LRRs assemble into a β-sheet that constitutes the concave surface of the entire SLRP core protein (**Figure 2**) (Scott et al., 2004). The core proteins of SLRPs are thought to carry a two-fold biological function in the skin: (1) Regulating collagen fibrillogenesis, fibril organization, and ECM assembly to control tissue strength and biomechanics (Rada et al., 1993), which is prerequisite for skin development (Smith and Melrose, 2015); (2) modulating the bioactivities of a myriad of cytokines, chemokines, ligands, and receptors (Tillgren et al., 2009; Chen and Birk, 2013; Hultgardh-Nilsson et al., 2015) that orchestrate the wound healing process (Barrientos et al., 2008). Besides, members of the SLRP family generally obtain GAG modifications post-translationally. The multitude of substitution sites on the SLRP core protein, along with variable glycosylation states, result in a variety of SLRPs which can further facilitate their interactions with various cell surface receptors, chemokines, cytokines, and growth factors (Kram et al., 2017). Abnormalities in SLPR expression or structure often alter matrix integrity and lead to dysfunctional matrix assembly in the skin, like those found in human pathological situations and SLRP-deficient animal models (Chen and Birk, 2013). For example, expression and structural changes of some SLRPs were noticed during skin development (Carrino et al., 2011), which was summarized below. Taken together, SLRPs are not only important for structural establishment of the ECM but also crucial in a variety of biological and pathological processes, such as the remodeling of the ECM during cutaneous injury and repair (Kalamajski and Oldberg, 2010; Karsdal et al., 2013; Tracy et al., 2016; Karamanou et al., 2018).

This review aims at summarizing all relevant available information about the spatial-temporal expression pattern of SLRPs in the skin, their relationship with human skin pathologies, and current understandings of their roles in skin wound healing to gain insights into their potentials as wound healing management pharmaceuticals.

## The SLRPs in Normal and Diseased Skin

Since decorin (DCN) was identified as the first SLRP (Krusius and Ruoslahti, 1986), many SLRP family members have been recognized in the last 30 years. Currently, 18 SLRPs have been divided into 5 classes based on the homologies at the genomic and protein level, the feature of the N-terminal cysteine residues with defined spacing, and chromosomal organization (Fisher et al., 1989; Henry et al., 2001; McEwan et al., 2006; Schaefer and Iozzo, 2008). For instance, SLRPs detected in the skin are predominantly Class I-III, which share a distinctive characteristic at the C-terminal, called an “ear” repeat (**Figure 2**). The “ear” repeat is the penultimate LRR that forms the most extended loop laterally from the convex face of the entire molecule (McEwan et al., 2006; Chen and Birk, 2011). The “ear” spreads from the first conserved C-terminal cysteine residue to the cysteine residue of the last LRR. Importantly, residues in the “ear” are not highly conserved among different SLRPs, which indicates a possible relationship to their specific functions. Consequently, the “ear” repeat is thought to maintain the configuration of the core protein and affect its ligand-binding ability (Chen and Birk, 2011; Chen and Birk, 2013). In comparison, Class IV and V SLRPs lack the “ear” repeat and are categorized as non-canonical classes of SLRPs (Schaefer and Iozzo, 2008).

### Class I SLRPs

Five SLRP members are identified in this class, including DCN (Danielson et al., 1997), biglycan (BGN) (Corsi et al., 2002), asporin (ASPN) (Henry et al., 2001), extracellular matrix protein 2 (ECM2) (Nishiu et al., 1998), and extracellular matrix protein X (ECMX) (Iozzo and Schaefer, 2015). DCN, BGN, and ASPN all present in the skin with different transcriptional patterns, and ECM2 mRNA expression can also be detected in the skin (Maquart et al., 2010).

#### Decorin (DCN)

Containing a 36 kDa core protein with single chondroitin sulfate (CS) or dermatan sulfate (DS) chain (Roughley, 2006), DCN has been considered as the predominant interstitial PG in human skin (Carrino et al., 2011; Li et al., 2013). In the skin, DCN has been detected mostly in the reticular dermis, but absent from the papillary dermis. Minor DCN expression was also found in the epidermis (Fleischmajer et al., 1991; Lochner et al., 2007). Since DCN comprises most of the type I collagen-binding PGs in human skin (Li et al., 2013), it is thought to play a critical role in the regulation of fibril structure in the skin.

In fetal rat skin, transcription of DCN increases between embryonic days 16.5 (E16.5) and E18.5 (term, 21.5 days) which is correlated to the transition from fetal-type scarless healing to adult-type scarring period in the skin (Soo et al., 2000; Zheng et al., 2016). In human skin, the level of DCN accelerates with aging (Carrino et al., 2000; Carrino et al., 2003). For example, a clinical study showed that the transcriptional level of DCN in skin biopsies from older adult donors (61–68 years) was twofold greater than that of their younger counterparts (25–35 years) (Lochner et al., 2007). In addition to its elevated expression, the molecular weight of DCN in older human skin was found to be significantly smaller due to the shortened GAG chains (Li et al., 2013). Similar results have been replicated in rats (Ito et al., 2001; Nomura et al., 2003). Importantly, the cutaneous wound healing process of the elderly is much slower, while all healing phases differ from their younger counterparts, including delayed inflammatory response, delayed proliferative response, and much weaker remodeling phase (Gerstein et al., 1993; Gould et al., 2015). Therefore, the elevated expression and reduced weight of DCN in the skin of the aged population may be associated with their functional alterations, although the underlying mechanism is not fully elucidated and warrants further investigations. Moreover, altered expression of DCN has been detected in a number of human diseases with skin phenotypes, including decreased DCN in fibroblasts in patients with neonatal Marfan syndrome (Raghunath et al., 1993), increased DCN in fibroblasts from patients with localized scleroderma (Izumi et al., 1995), and increased DCN in fibroblasts from patients with systemic sclerosis (Westergren-Thorsson et al., 1996). Targeted disruption of DCN in mice results in abnormal collagen fibril morphology and skin fragility with markedly reduced tensile strength (Danielson et al., 1997). Another study showed that DCN and BGN double-knockout (KO) mice directly resemble the rare progeroid variant of human Ehlers-Danlos syndrome (EDS), in which skin fragility and progeroid changes in the skin (reduced hypodermis) are dramatically displayed (Corsi et al., 2002). Furthermore, in a progeroid patient carrying two point mutations in beta-1,4-galactosyltransferase 7 (B4GALT7), only 50% of the DCN exhibit GAG side-chain substitution on their core protein, which is thought to be a major mechanistic cause for the skin and wound healing defects observed in this patient with the progeroid form of EDS (Gotte and Kresse, 2005). These investigations indicate that DCN is crucial to the normal function of the skin and maybe a potential candidate for pharmacological development in the treatment of some skin diseases.

#### Biglycan (BGN)

Another well-studied Class I SLRP is BGN. BGN usually contains a 38 kDa core protein attached with two CS/DS chains; however, nonglycanated forms of BGN have also been detected in human intervertebral discs (Johnstone et al., 1993). While both BGN and DCN belong to Class I SLRPs and are able to bind with type I collagen fibrils directly, their spatial expression in skin is very different. For instance, DCN is mainly synthesized by interstitial fibroblasts, whereas BGN is secreted by both dermal and epidermal cells (Li et al., 2013). Besides, BGN is present in the connective tissue sheath of the hair follicle (Malgouries et al., 2008).

Similar to DCN, decreased BGN expression was also found in fibroblasts isolated from the skin of systemic sclerosis patients (Westergren-Thorsson et al., 1996). Although BGN deficiency in mice also induces changes in collagen fibril morphology in the skin and leads to the mild cutaneous abnormalities with thinning of the dermis, skin fragility of BGN-deficient mice is not noticeably altered (Corsi et al., 2002). Corsi et al. claimed that the skin abnormalities in BGN-deficient mice were more subtle in comparison with DCN-deficient mice (Corsi et al., 2002), which in turn resulted in that research was not focused on BGN and its role in skin and wound healing.

#### Asporin (ASPN)

Unlike general SLPRs, ASPN contains a 43 kDa core protein, but lacks a GAG side chain (Lorenzo et al., 2001) and carries a polymorphic calcium-binding polyaspartate sequence (Kalamajski et al., 2009). ASPN has been found in dermis, perichondrium and periosteum, tendon, and eye sclera (Kou et al., 2007). ASPN-null mice exhibit an increased skin mechanical toughness due to the altered GAG composition and structure in the ECM (Maccarana et al., 2017). However, ASPN has not been studied in depth for its involvement in skin development and cutaneous wound healing.

### Class II SLRPs

To date, this class contains 5 members that can be divided into 3 subgroups based on their protein homology. Subgroup A consists of fibromodulin (FMOD) (Velez-Delvalle et al., 2008) and lumican (LUM) (Yeh et al., 2010), subgroup B includes keratocan (KERA) (Corpuz et al., 1996) and proline and arginine rich end leucine rich repeat protein (PRELP) (Grover and Roughley, 2001), and subgroup C is comprised of osteomodulin (OMD) (Tasheva et al., 2002).

#### Fibromodulin (FMOD)

FMOD has a 42 kDa core protein with up to 4 N-linked keratan sulfate (KS) attached, which shares significant sequence homology with DCN and BGN (Antonsson et al., 1993). In the skin, FMOD is predominately secreted by dermal fibroblast and is also expressed by human epidermal keratinocytes *in vitro* and detected in the human epidermis *in vivo* (Velez-Delvalle et al., 2008).

Unlike other SLRPs, expression of FMOD significantly decreases during the transition from fetal-type scarless repair to adult-type repair with scaring in a fetal rat skin model (Soo et al., 2000; Zheng et al., 2016). Moreover, our recent study demonstrated that FMOD is essential for fetal-type scarless cutaneous wound healing by loss- and gain-of-function studies in mouse and rat models (Soo et al., 2000; Zheng et al., 2016).

Although FMOD-null mice showed no apparent defects in the unwounded skin (Chakravarti, 2002), a wider distribution of collagen fibril diameters accompanied with enlarged interfibrillar spaces between collagen fibrils was observed (Khorasani et al., 2011). Meanwhile, thinner collagen fibrils and abnormal fibers with increased deposition of LUM were also found in the tendons of FMOD-null mice (Svensson et al., 1999). As expected, FMOD and LUM double-deficient mice showed more obvious abnormalities, such as reduced body size, increased skin hyperextensibility, escalated gait abnormality, intensified joint laxity, and accelerated age-dependent osteoarthritis resembling EDS (Jepsen et al., 2002). These abnormal phenotypes may indicate a functional overlap between FMOD and LUM in modulating the ECM and cellular behavior in a broad range of tissues (Chakravarti, 2002; Jepsen et al., 2002). It is known that the re-organization of ECM is necessary during the healing process since pathological scarring occurs when the ECM is not appropriately reformed. Thus, the fact that FMOD is essential for regular collagen fibril organization in connective tissues suggests that FMOD may play a pivotal role in skin wound healing.

#### Lumican (LUM)

LUM was first isolated from the chicken cornea (Blochberger et al., 1992). LUM has a 38 kDa core protein with 4 N-linked sites within the LRR domain of the core protein that can be substituted by KS (Scott, 1996). It is expressed in the subepithelial dermis by dermal fibroblasts (Ying et al., 1997; Chakravarti et al., 1998). Interestingly, LUM is also secreted by melanoma cells but not normal melanocytes (Sifaki et al., 2006).

Unlike FMOD whose expression is reduced from early/mid-gestation when skin wounds heal scarlessly to late-gestation when skin wounds end up with adult-type scarring, LUM expression in fetal skin is upregulated during the same transition period, much like DCN (Zheng et al., 2016). On the contrary, a significant negative correlation between LUM transcriptional levels in human skin fibroblasts and donors’ age was observed in a study involving 1-month- to 83-year-old participants (Vuillermoz et al., 2005). The steady decline in LUM expression accompanied by the upregulation of DCN expression with aging indicates that these changes may be contributing to the functional impairment of fibroblasts during aging, such as decreased fibroblast growth and survival (Campisi, 1998; Brown, 2004; Vuillermoz et al., 2005). Interestingly, similar to DCN-deficient mice, LUM-null mice display skin laxity and fragility resembling EDS (Chakravarti et al., 1998). It is worth noting that wounds in FMOD-null mice have delayed dermal fibroblast migration but accelerated epidermal migration accompanied by elevated LUM expression (Zheng et al., 2014b), indicating FMOD and LUM may predominately function on fibroblast and keratinocytes, respectively. Thus, in comparison with FMOD whose biopotency is mainly assessed on dermal functions (Zheng et al., 2014a; Zheng et al., 2014b; Zheng et al., 2016), the investigation of LUM is more focused on the cornea in which epidermal migration plays more essential roles during wound healing (Saika et al., 2000; Seomun and Joo, 2008; Frikeche et al., 2016).

#### Keratocan (KERA)

KERA is a 60–70 kDa KS substituted member of the SLRP family (Mary and James, 2015). It is mainly abundant in the cornea and detected in much lesser amount in the skin as a non-sulfated glycoprotein (Corpuz et al., 1996). The variety of the abundance and GAG structure of KERA found in different tissues suggests that its function be tissue-dependent. For example, in the cornea, KERA with long, highly sulfated KS chains has been thought to be essential for corneal transparency (Kao and Liu, 2002). Thus, similar to LUM (Yamanaka et al., 2013), KERA is also a potential target for cornea healing therapies (Kao and Liu, 2002; Carlson et al., 2003; Liu et al., 2003; Chen et al., 2011). However, its role in the skin is still ambiguous for delineating.

#### Proline and Arginine Rich End Leucine Rich Repeat Protein (PRELP)

PRELP contains a 55-kDa core protein with no GAG and an N-terminal region which is highly unique, conserved, and rich in arginine and proline (Bengtsson et al., 2000). It functions as a molecule by anchoring basal membranes to the underlying connective tissue (Grover and Roughley, 1998; Grover and Roughley, 2001). For example, PRELP is expressed in the basement membrane between the epidermis and the dermis in the skin (Bengtsson et al., 2002). Overexpression of PRELP in mice leads to reduced collagen fiber bundle content and size in the dermis and decreases the thickness of the hypodermal fat layer in the skin (Grover et al., 2007), which somewhat resembles the symptoms of Hutchinson–Gilford progeria (Mounkes et al., 2003). In addition, PRELP can bind to perlecan (Bengtsson et al., 2002), which is thought to be essential for epidermal formation by regulating the survival of keratinocytes (Sher et al., 2006). This indicates that PRELP may participate in regulating the function of keratinocytes, but further studies are needed to elucidate it. However, a clear application of PRELP for wound healing is still lacking.

Interestingly, in comparison with Class I SLRPs, most Class II SLRPs seem to have a more executive function on epidermal keratinocytes. The one exception is FMOD, which has proven to be critical for maintaining the normal function of dermal fibroblasts (Zheng et al., 2016), as well as endothelial cells (Adini et al., 2014; Zheng et al., 2014a), like DCN and BGN. The response to different cell types may pave the fundamental for developing combination therapies of SLRPs to target both dermal and epidermal cells simultaneously to maximize their complementary biopotency and thus to optimize the skin wound healing outcome.

### Class III SLRPs

To date, osteoglycin (OGN, also known as mimecan) (Tasheva et al., 2002), epiphycan (EPYC) (Johnson et al., 1997) and opticin (OPTC) (Reardon et al., 2000) constitute this class, which is characterized by a relatively low number of LRRs (7 LRRs) compared to the classic 10-12 LRRs of other classes.

OGN was first identified as a 25 kDa KS SLRP in the cornea, and a 36 kDa OGN protein without KS chains was also detected in other connective tissues including aorta, sclera, skin, cartilage, the vagus nerve, and in lesser amounts in the cerebellum, kidney, intestines, myocardium, and skeletal muscle (Funderburgh et al., 1997). As for its role in the skin, OGN-deficient mice display skin with moderately reduced tensile strength, which is correlated to the presence of thicker collagen fibrils that possess marked increases in collagen fibril diameter. OGN also plays a pivotal role in collagen fibrillogenesis in the skin (Tasheva et al., 2002). Although transcription of EPYC and OPTC have been detected in the skin (Reardon et al., 2000; Takanosu et al., 2001; Maquart et al., 2010), their biological function in the skin is unclear.

### Class IV and Class V SLRPs

Class IV and V SLRPs are considered to be non-canonical classes of SLRPs. These include chondroadherin (CHAD) (Haglund et al., 2011), nyctalopin (NYX) (Bech-Hansen et al., 2000), tsukushi (TSKU) (Ohta et al., 2004), podocan (PODN) (Ross et al., 2003), and recently identified podocan like 1 (PODNL1) (Mochida et al., 2011). The function and spatial-temporal expression patterns of these SLRPs in the skin are rarely studied. A previous study detected CHAD mRNA in keratinocytes, and NYX mRNA in keratinocytes and skin fibroblasts (Maquart et al., 2010). Future studies, not necessarily limited to the skin, are required to reveal their biological functions.

The expression and distribution of known SLRPs in the skin, as well as the abnormalities observed in SLRP-deficient mice, are summarized in **Table 1**.

**Table 1:** The expression and distribution of SLRPs and abnormalities of knock out and overexpression mice in the skin.

SLRP	Expression and distribution in the skin	Mice model	Abnormalities in the skin
Decorin	Most expression in the dermis and minor expression in the epidermis	Targeted disruption of decorin in exon2	Skin fragility with markedly reduced tensile strength
Biglycan	Expression in the dermis, the epidermis and the sheath of hair follicle	Targeted disruption of biglycan in exon2	Mild skin abnormalities with thinning of the dermis but without distinct skin fragility
Decorin and Biglycan	Not applicable	Decorin and biglycan double-knockout mice	Skin fragility and progeroid changes in the skin (reduced hypodermis)
Asporin	Expression in the dermis	Targeted disruption of asporin in exons 2–3	Increased skin mechanical toughness
Fibromodulin	Expression in the dermal fibroblast and human epidermal keratinocytes *in vitro* and the epidermis *in vivo*	Targeted disruption of fibromodulin in exon2	No overt defects in skin, but larger collagen fibrils and less orderly packed collagen fibrils with increased interfibrillar space
Lumican	Expression in the dermis	Targeted disruption of lumican in exon2	Skin laxity and fragility
Fibromodulin and Lumican	Not applicable	Fibromodulin and lumican double-knockout mice	Additional gross skin phenotypes including skin hyperextensibility
PRELP	Expression in the basement membrane between the epidermis and the dermis	Overexpression of PRELP transgenic mice	Decreased collagen fiber bundle content and size in the dermis, and the thinner hypodermal fat layer
Osteoglycin	Expression in the skin	Targeted disruption of osteoglycin in exon2	Reduction in the tensile strength of the skin, thicker collagen fibrils and a significant increase in collagen fibril diameter in the skin

## The SLRPs in Skin Wounds and Wound Healing

As a protective barrier shielding the human body from the environment, the skin plays a pivotal role in maintaining physiological homeostasis of the human body. Any lesion breaking the skin barrier will make the organism vulnerable to infections, thermal disorders, and fluid loss (Sorg et al., 2017). Skin wound healing is a dynamic, complex and tightly regulated process comprised of hemostasis, inflammation, proliferation, remodeling, and maturation phases, in which various cell types and mediators are recruited at the wound site, and complex interactions exist between different cells and the ECM (Martin, 1997; Diegelmann and Evans, 2004; Gibran et al., 2007; Artlett, 2013). During the process of wound healing, the ECM not only provides structural support for the tissues, but also serves as a platform for cells and mediators that regulates inter/intracellular signaling (Ghatak et al., 2015). As essential components of the ECMs, many SLRPs participate in a diversity of signaling pathways to regulate cellular activities during the wound healing process.

### Inflammation

Following an injury, skin cells are exposed to acute inflammatory signals such as pathogen-associated molecular patterns (PAMPs) or damage-associated molecular patterns (DAMPs) (Takeuchi and Akira, 2010; Strbo et al., 2014). These patterns can be recognized by toll-like receptors (TLRs) to initiate inflammation. Leukocytes are attracted to the site of injury, accompanied by elevated levels of pro-inflammatory cytokines, and thus amplify the inflammatory response (Eming et al., 2014; Vestweber, 2015). Gradually, macrophages will display a transition from the M1 subset (phagocytic activity and production of pro-inflammatory cytokines) (Galli et al., 2011; Sindrilaru and Scharffetter-Kochanek, 2013) to the M2 subset (reparative activity with the synthesis of anti-inflammatory mediators and the production of the ECM) (Brancato and Albina, 2011; Sindrilaru and Scharffetter-Kochanek, 2013). This switch corresponds to the transition from the inflammation stage to the proliferation stage in the wound healing process.

Although there have been studies investigating the effects of SLRPs on the inflammatory response, most do not specifically focus on skin wound healing. Several studies have shown that DCN, BGN, and LUM can interact with TLR2 and/or TLR4 signaling pathways in innate immune responses to combat microbial pathogens. For instance, in mouse peritoneal macrophages, DCN induces tissue necrosis factor (TNF) and programmed cell death 4 (PDCD4) production through TLR2 and TLR4, which enhances the proinflammatory effects of lipopolysaccharides (LPS), a vital constituent of Gram-negative bacteria, which can trigger a robust immune response (Merline et al., 2011). BGN has also been proven to be a proinflammatory factor in mouse peritoneal macrophages by regulating the same signal pathways as DCN (Schaefer et al., 2005). Like DCN and BGN, LUM enhances host immune responses to LPS *via* TLR4 in mouse peritoneal macrophages (Wu et al., 2007). Unsurprisingly, LUM-null mice are hypo-responsive to LPS-induced septic shock with reduced pro-inflammatory cytokines production (Wu et al., 2007). Also, LUM has been shown to regulate inflammation in the development of colitis in mice (Lohr et al., 2012), and accelerate LPS-induced renal injury in mice *via* TLR4-nuclear factor κB (NFκB) pathway (Lu et al., 2015). Moreover, in LPS-induced wounds of the cornea, no induction of TNF or IL1β, and reduced infiltration of neutrophils and macrophages were found in LUM-null mice (Vij et al., 2005).

In contrast to their pro-inflammatory functions in the aforementioned infection scenario, SLRPs may act as anti-inflammatory factors to inhibit excessive inflammation during wound healing in the skin. For example, FMOD-null mice exhibit elevated and prolonged inflammatory infiltration in the skin wound area, accompanied by delayed reepithelialization (Zheng et al., 2014b). Similarly, LUM-deficient mice display an increased inflammatory macrophage density with delayed cutaneous wound healing (Yeh et al., 2010). Furthermore, TSKU has been detected in fibroblasts, myofibroblasts, and macrophages during skin wound healing in mice. Likewise, loss of TSKU causes increased TGFβ1 expression and excess inflammation (Niimori et al., 2014). Collectively, these studies suggest that SLRPs may play a diverse role in inflammatory response regulation, which may highly depend on the microenvironment.

### Angiogenesis

The process of angiogenesis occurs accompanied by fibroblast proliferation when endothelial cells migrate to the wound site and provide the nutritive perfusion for fibroblasts and epithelial cells during the healing process (Martin, 1997; Demidova-Rice et al., 2012; Sorg et al., 2017). The involvement of SLRPs has been identified in the angiogenesis of wound healing, tumorigenesis, and other inflammatory processes. For instance, DCN exhibits antiangiogenic activities during cutaneous wound healing, while higher DCN expression was detected in human benign tumors *vs.* malignant vascular tumors (Jarvelainen et al., 2006; Salomaki et al., 2008). Meanwhile, impaired angiogenesis was found in the injured cornea of DCN-null mice (Schonherr et al., 2004), and repressed angiogenesis was also present in some tumors associated with reduced DCN expression (Nayak et al., 2013; Chui et al., 2014). These studies suggest that DCN can be either stimulatory or suppressive for angiogenesis, which may be related to the physiologic and pathologic conditions of tissues (Järveläinen et al., 2015).

BGN has been shown to have a proangiogenic effect in fracture healing (Berendsen et al., 2014; Myren et al., 2016), colon cancer (Xing et al., 2015), and tumor endothelial cells (Yamamoto et al., 2012), though its role in angiogenesis during skin wound healing is not clear.

FMOD was found to promote angiogenesis during cutaneous wound healing (Zheng et al., 2011; Zheng et al., 2014a). Particularly, FMOD was found to accelerate human umbilical vein endothelial cell adhesion and spreading, actin stress fiber formation, and eventually tube-like structure network establishment *in vitro* (Jian et al., 2013). Furthermore, it has been confirmed that FMOD stimulates angiogenesis in various *in vivo* systems, such as neovascularization, wound healing and Matrigel™ plug assays. FMOD was also found to enhance vascular sprouting during normal retinal development (Adini et al., 2014). FMOD also promotes tumor angiogenesis of small cell lung cancer by upregulating angiogenic factor expression (Ao et al., 2017). Overall, these studies constitute evidence that FMOD displays angiogenic biopotency in numerous biological processes.

On the other hand, LUM was found to inhibit angiogenesis by interfering with integrin α2β1 activity and repressing matrix metalloproteinase (MMP)14 expression *in vivo* (Niewiarowska et al., 2011). LUM has also been identified as an inhibitor for tumor angiogenesis (Albig et al., 2007; Brezillon et al., 2009; Williams et al., 2011). Interestingly, angiogenesis was not altered in LUM-deficient mice in aortic ring assays, Matrigel™ plugs, or healing wound biopsies (Sharma et al., 2013). Thus, LUM is thought to exhibit an antiangiogenic effect in restricted circumstances, possibly only in some specific tumor microenvironments.

In summary, these studies paint the picture of SLRPs playing a wide range of roles in the angiogenesis of various biological processes. Specifically, FMOD is the only SLRP confirmed to enhance angiogenesis during skin wound healing, suggesting that it may have therapeutic potential in cutaneous healing of poorly vascularized wounds, such as in the scenarios of diabetic wounds.

### Fibroblast Activities

Dermal fibroblasts are the predominant cellular component in the wound healing process. During the proliferation stage, fibroblasts migrate into the wound site, and gradually grow to produce a new provisional ECM through the production of collagen and fibronectin (Midwood et al., 2004). Wound contraction will occur when fibroblasts differentiate into myofibroblasts after reepithelialization. This process decreases the size of the wound and is followed by the removal of unneeded cells through apoptosis (Hinz, 2006). SLRPs are known to impact several of the critical functions of fibroblasts during wound healing, including migration, proliferation, differentiation, and collagen synthesis. As a result, abnormal expression of SLRPs can disrupt the wound healing process and possibly result in pathological scarring, as seen in keloids and hypertrophic scars.

**Figure 3:**
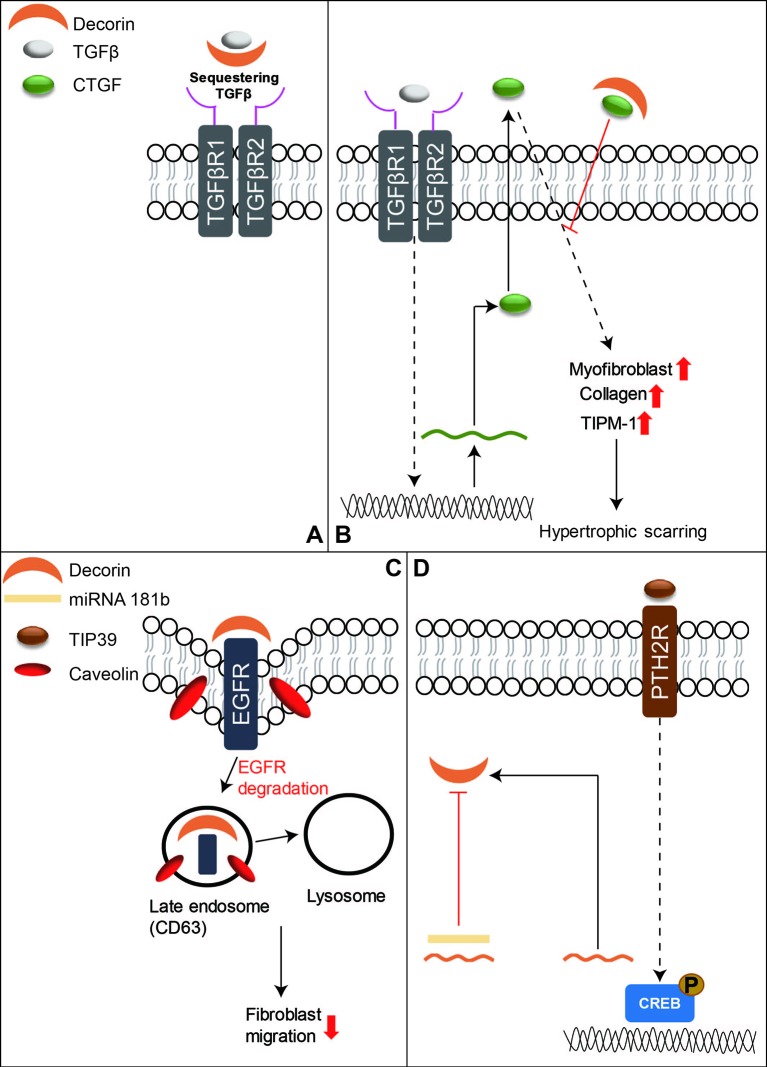
A schematic diagram of the functions of DCN in skin wound healing. **(A)** DCN is known to bind to mammal TGFβ isoforms to sequester their signal transduction. **(B)** DCN can also bind to CTGF, which is a downstream mediator of TGFβ1 signaling, to reduce hypertrophic scarring. **(C)** In addition, DCN can repress fibroblasts migration by inducing EGFR degradation. **(D)** On the other hand, activation of TIP39-PTH2R signaling or blocking of microRNA 181b can induce DCN production, and thus benefit skin wound repair.

TGFβ signaling has been thought to play a central role in both skin wound healing and scar formation (Faler et al., 2006; Penn et al., 2012; Pakyari et al., 2013), and DCN is known to bind to all three mammal TGFβ isoforms and represses their activity by sequestering the isoforms to the ECM and thus inhibiting their signal transduction (**Figure 3A**) (Droguett et al., 2006; Penn et al., 2012). DCN was also found to interact with CTGF-a downstream mediator of TGFβ1 signaling (**Figure 3B**) (Daniels et al., 2003; Shi-Wen et al., 2008; Vial et al., 2011). Moreover, DCN is able to activate epidermal growth factor receptor (EGFR)‐mediated receptor auto-phosphorylation and downstream signaling pathways, such as the mitogen-activated protein kinase (MARK)1/3 pathway, to mobilize intracellular calcium, and activate other EGFR‐dependent pathways in tumor cells to suppress cell growth (Moscatello et al., 1998; Patel et al., 1998). DCN displays similar cell growth suppression ability in dermal fibroblasts (Laine et al., 2000; Tran et al., 2004). Furthermore, a recent study revealed that DCN repressed corneal stromal fibroblasts migration *via* inducing EGFR degradation (**Figure 3C**) (Mohan et al., 2019), which has not been well investigated in the context of skin wounds, although low levels of DCN and increased activation of the MARK1/3 signaling have been observed in keloid tissues (Meenakshi et al., 2009). In addition to regulating growth factor signaling transduction, DCN serves as a stabilizer of the ECM tissue structure through binding of type I collagen and thus downregulates cellular proliferation and migration, as well as protein synthesis in a number of biological and pathologic processes (Iozzo, 1999; Tran et al., 2004). Keloid fibroblasts have less DCN expression than normal fibroblasts (Mukhopadhyay et al., 2010) while forcing DCN expression by adenovirus in keloid fibroblasts remarkably reduced their collagen synthesis and upregulated the transcriptional level of MMP1 and MMP3 (Lee et al., 2015). The expression of DCN in the fibroblasts isolated from the deep dermis was also lower than those derived from the superficial dermis (Honardoust et al., 2012b). This phenomenon indicates a possible relationship between lower DCN expression and hypertrophic scarring as deep dermal injuries often lead to hypertrophic scarring while superficial cutaneous wounds usually heal with minimal scarring (Wang et al., 2008; Marshall et al., 2018). In comparison to unwounded skin, post-burn hypertrophic scar tissue also has a lower level of DCN (Scott et al., 1995; Sayani et al., 2000). While DCN-deficient mice exhibit significantly postponed cutaneous wound healing (Jarvelainen et al., 2006). The lower DCN levels in keloids and hypertrophic scars may contribute to the unordered collagen arrangement and excess ECM production. Meanwhile, recombinant human DCN inhibits fibroblast proliferation and downregulates TGFβ1 production, and collagen synthesis in hypertrophic scar fibroblasts (Zhang et al., 2007). Moreover, DCN can inhibit the contraction of collagen gel encapsulation in normal or hypertrophic scar fibroblasts, which gives further indication that it may pose therapeutic potential in hypertrophic scarring (Zhang et al., 2009). Recent studies show that activation of Tuberoinfundibular peptide of 39 residues (TIP39)—Parathyroid Hormone 2 Receptor (PTH2R) signaling or blocking of microRNA 181b can induce DCN expression and promote wound repair (**Figure 3D**) (Kwan et al., 2015; Sato et al., 2017). Additionally, a collagen-binding peptidoglycan derived from DCN has been shown to inhibit MMP-mediated collagen degradation *in vitro* and reduce scar formation in mice (Stuart et al., 2011). Collectively, these results indicate that DCN may be a potential therapeutic agent for keloids and hypertrophic scars.

As a structurally homologous protein of DCN, BGN can also bind to TGFβ isoforms to attenuate its signal transduction (Hildebrand et al., 1994; Droguett et al., 2006; Penn et al., 2012). Expression of BGN was not altered in excisional skin wounds and hypercontracted/hyperpigmented scarring pig models (Olson et al., 2000; Gallant et al., 2004). However, the upregulation of BGN was observed in adult rat wound healing models (Soo et al., 2000). Elevated BGN expression was also observed in hypertrophic scars compared with that in normal scars (Armour et al., 2007; Honardoust et al., 2011). Deep dermal fibroblasts also have a higher BGN level than that of the superficial dermal fibroblasts (Honardoust et al., 2011; Honardoust et al., 2012a). Unfortunately, whether higher BGN expression in deep dermal fibroblasts is relevant to the profibrotic or inflammatory response in deep dermal cutaneous injuries remains elusive. Although BGN has been shown to regulate proinflammatory cytokine expression and inflammatory response by TLR2 and TLR4 in the kidney, lung, and circulation (Babelova et al., 2009). Moreover, BGN transcription was up-regulated in keloid tissues (Hunzelmann et al., 1996). Interestingly, basic fibroblast growth factor (bFGF) can up-regulate BGN while suppressing DCN expression in keloid fibroblasts (Tan et al., 1993). Taken together, these results imply that BGN may be related to keloid and hypertrophic scarring. Further investigations are needed to confirm the involvement of BGN in cutaneous wound healing and elucidate the specific roles of BGN and DCN during the scar formation.

FMOD was down-regulated during adult rat wound healing with scar formation (Soo et al., 2000). Importantly, loss of FMOD can eliminate the ability of early-gestation fetal rodents to heal without scarring. Meanwhile, the administration of FMOD alone was capable of restoring scarless healing in late-gestation rat fetal wounds, which would naturally heal with scar (Zheng et al., 2016). In addition to restoring scarless wound healing in late-gestation fetal wounds, forcing FMOD elevation by adenovirus can also promote skin wound healing in adult rabbit full-thickness incisions (Stoff et al., 2007). Additionally, FMOD-deficient mice exhibit delayed wound closure and increased scar formation (Zheng et al., 2011; Zheng et al., 2014b).

**Figure 4:**
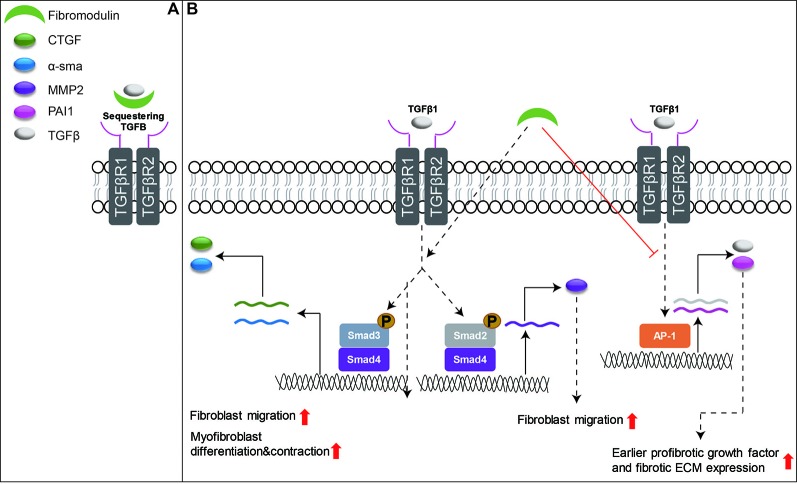
Schematic depiction of the functions of FMOD in skin wound healing. **(A)** FMOD is able to bind to all three mammal TGFβ isoforms as an extracellular TGFβ reservoir. **(B)** Importantly, FMOD can selectively enhance SMAD3-mediated TGFβ1-responsive pro-migration and pro-contraction signaling, while reducing AP-1-mediated TGFβ1 auto-induction and fibrotic ECM accumulation during adult cutaneous wound healing.

Many review articles have already focused on the essential role of TGFβ signaling in wound healing (Faler et al., 2006; Penn et al., 2012; Pakyari et al., 2013; Lichtman et al., 2016). However, among SLRPs, only FMOD has been studied in detail about its interaction with TGFβ signaling to orchestrate the function of fibroblasts to enhance skin wound healing (Zheng et al., 2017). For example, the delayed cutaneous wound closure in FMOD-deficient mice may be attributed to the elevated local TGFβ3 levels (Zheng et al., 2011), since TGFβ3 selectively postpones dermal fibroblast proliferation and migration into the wound area (Bandyopadhyay et al., 2006; Han et al., 2012). Moreover, adult FMOD-null mouse wounds have higher expression of TGFβ receptors in comparison with their wild-type counterparts during the proliferative stage, but reduced expression of TGFβ ligands and receptors during the remodeling stage (Zheng et al., 2014b). Similar to DCN, FMOD is downregulated in postburn hypertrophic scars (Honardoust et al., 2011). Reduced fibromodulin in the deep dermis of the skin is thought to contribute to the development of hypertrophic scars after injuries (Honardoust et al., 2012a; Honardoust et al., 2012b). Interestingly, FMOD transcription was not altered following wound creation in an adult Yorkshire pig model, but exhibited a biphasic pattern of mRNA expression (initial increased at day 14, followed by decreased levels at days 28–42 and then a second peak by days 56–70) in an adult red Duroc pig model (Olson et al., 2000; Gallant et al., 2004). These observations are aligned with the previous hypothesis that the healing profile of the red Duroc pig wound model (which simulates hypertrophic healing in humans) (Harunari et al., 2006; Xie et al., 2007; Zhu et al., 2007) is inherently different from that of the Yorkshire pig wound model (which simulates normal scarring) (De Hemptinne et al., 2008; Seaton et al., 2015). Mechanically, like DCN and BGN, FMOD shows similar properties in its ability to bind to mammal TGFβ isoforms; however, it is a more effective competitor for TGFβ binding than DCN or BGN (Hildebrand et al., 1994). Traditionally, FMOD was considered an extracellular TGFβ reservoir (**Figure 4A**). Our recent studies have deeply explored the mechanisms by which FMOD orchestrates TGFβ1 signaling and subsequently reduces scar formation in adult skin wounds (Zheng et al., 2017): “(1) like fetal wounds (Larson et al., 2010), FMOD treatment to adult wounds causes reduced and more transient TGFβ1 expression; (2) like fetal wounds (Walraven et al., 2015), FMOD treatment induces high level of SMAD2 and SMAD3 phosphorylation, and low levels of several fibrosis-associated targets; (3) like fetal fibroblasts (Sandulache et al., 2007), FMOD treatment results in a more migratory and contractile phenotype; (4) like fetal fibroblasts (Colwell et al., 2006), FMOD treatment exhibits higher TGFβ1-stimulated CTGF expression levels for increased myofibroblast differentiation and contraction; and (5) much like fetal wounds (Cass et al., 1997), FMOD treatment results in more rapid myofibroblast clearance from the wound” (**Figure 4B**). Taken together, FMOD administration in adult wound models elicits a similar phenotype to fetal wounds at the molecular, cellular, and gross morphological levels (Zheng et al., 2017). Moreover, recent studies demonstrate that FMOD can directly reprogram human dermal fibroblasts into a multipotent stage, indicating its ability to regulate the intracellular signaling cascade and determine the cell fate (Zheng et al., 2012; Li et al., 2016; Zheng et al., 2019). Furthermore, from a translational aspect, recent studies have confirmed the biopotency of FMOD in reducing scar formation, accelerating wound tensile strength reestablishment, and improving dermal collagen architecture organization as well as gross wound appearance in multiple small and large preclinical animal models, and even within an excessive-mechanical-loading model (Zheng et al., 2017; Jiang et al., 2018), highlighting the enormous potentials of FMOD as a regenerative medicine for wound and scar therapies. Encouragingly, we have developed an FMOD-derived peptide (SLI-F06) which is being tested in a clinical trial for optimizing cutaneous wound healing (clinicaltrials.gov: NCT03880058).

**Figure 5:**
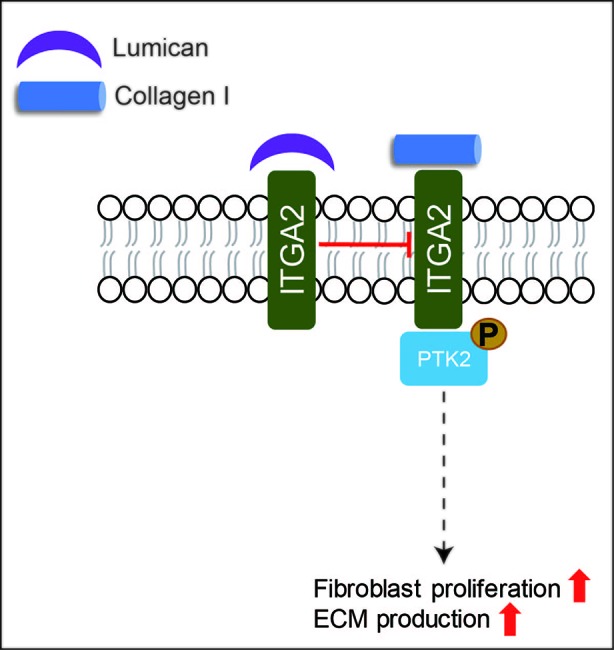
A schematic description of the functions of LUM in skin wound healing. LUM can suppress excessive fibroblast proliferation and ECM production *via* inhibiting ITGA-PTK2 signaling through binding to ITGA2.

LUM is the only known SLRP expressed by the epithelia during wound healing (Frikeche et al., 2016; Karamanou et al., 2018). During cornea wound healing, LUM is known to regulate collagen fibrillogenesis, keratinocyte phenotypes, corneal transparency modulation, angiogenesis, and extravasation of inflammatory cells (Park and Tseng, 2000; Vij et al., 2005; Hayashi et al., 2010; Chen et al., 2011; Karamanou et al., 2018). However, the role of LUM in skin wound healing has not been adequately assessed. Liu et al. reported that recombinant LUM protein promoted skin wound healing in adult mice by facilitating dermal fibroblast activation and contraction without promoting keratinocyte proliferation and migration (Liu et al., 2013). The increased fibroblast contractibility induced by LUM is regulated by integrin subunit alpha (ITGA)-2 (Liu et al., 2013). Meanwhile, Zhao et al. reported that adenovirus-mediated LUM-overexpression suppressed excessive fibroblast proliferation and ECM production *in vitro via* inhibiting collagen - ITGA2 - protein tyrosine kinase (PTK)2 signaling through binding to the collagen receptor ITGA2 (**Figure 5**), which in turn to reduce scar formation by significantly inhibiting ECM deposition *in vivo* (Zhao et al., 2016). More interestingly, in comparison with normal skin-derived fibroblasts, hypertrophic scar-derived fibroblasts displayed reduced LUM expression (Zhao et al., 2016), while keloid-derived fibroblasts exhibited elevated LUM expression (Naitoh et al., 2005). Although LUM may be a potential pharmaceutical candidate for skin wound healing, its mechanism of action is far from clear.

ASPN is upregulated in keloid margin biopsy specimens compared with that from adjacent healthy skin, indicating it may serve as a potential biomarker for keloid disease (Shih et al., 2010). Likewise, comparative mass spectrometry-based proteomic analysis of keloids and healthy skin has shown that ASPN expression was significantly increased in keloid scars. This suggests that ASPN may be potentially used as a specific target for therapeutic intervention (Ong et al., 2010). In addition, upregulated OGN expression was also found in keloids by cDNA microarray analysis (Naitoh et al., 2005). However, except for DCN, BGN, FMOD, and LUM, investigations into other SLRPs for their potential benefits in skin wound healing are still very much in their infancy.

## Conclusion and Perspectives

SLRPs are important components of the ECM that play crucial roles in collagen fibril growth, fibril organization, and ECM assembly. They are also capable of modulating the function of a significant number of growth factors and cytokines and have even been thought to prevent fibrosis and organ dysfunction (Mecham, 2011; Schaefer, 2011; Klingberg et al., 2013). Thanks to worldwide collaboration over the last 30 years, much has been discovered in regard to the pivotal roles that SLRPs play in the different phases of the skin wound healing process, as well as the therapeutic potentials of SLRPs for reducing scar formation. However, the precise details concerning how each individual SLRP functions in the different phases of the healing process are still unclear. It is crucial to gain a specific understanding of the nature of the SLRP functional components, particularly in regard to their interactions with cell surface receptors, growth factors, and the ligands associated with molecular patterns of the skin wound healing process.

In conclusion, although there are still many obstacles that need to be surmounted before SLRPs can be applied in clinics for cutaneous wound healing management, a variety of SLRPs, such as DCN, BGN, FMOD, and LUM, exhibit great potential for future use in skin wound healing. For example, given the pro-angiogenic, pro-migratory, and pro-contraction potential of FMOD (Zheng et al., 2014a; Zheng et al., 2014b; Zheng et al., 2017), besides reducing scar formation, it may be used to accelerate the healing of wound with retarded closure such as seen in the diabetic patients (Falanga, 2005; Ekmektzoglou and Zografos, 2006). Considering LUM enhances the epithelial cell migration (Seomun and Joo, 2008), it could also be a candidate to address the major problem of delayed wound healing. In addition to the skin, LUM and KERA may also be beneficial for corneal wound healing (Saika et al., 2000; Carlson et al., 2005), such as in the scene of corneal wounds caused by refractive surgeries (Ljubimov and Saghizadeh, 2015). Nevertheless, the delivery system and administration regimen of these SLRPs are insurmountable issues to be optimized in the pursuit of the most promising outcomes.

## Author Contributions

ZZ conceived the presented idea. XP wrote the manuscript with support from ZZ. ND modified the grammar and improved the language. All authors contributed to the final manuscript.

## Conflict of Interest

ZZ is an inventor on fibromodulin-related patents assigned to UCLA, and he is also a founder and former officer of Scarless Laboratories, Inc., which sublicenses fibromodulin-related patents from the UC Regents, who also holds equity in the company.

The remaining authors declare that the research was conducted in the absence of any commercial or financial relationships that could be construed as a potential conflict of interest.
